# Enhancing clinical utility of AI-based antimicrobial resistance models: a perspective

**DOI:** 10.1128/mbio.00776-26

**Published:** 2026-06-17

**Authors:** Robert A. Bonomo, Andrea M. Hujer

**Affiliations:** 1 Research Service, Louis Stokes Cleveland Department of Veterans Affairs Medical Center, VA Northeast Ohio Healthcare System 465630, Cleveland, Ohio, USA; 2 Department of Medicine, Case Western Reserve University School of Medicine 12304, Cleveland, Ohio, USA; 3 Department of Molecular Biology and Microbiology, Case Western Reserve University School of Medicine 12304, Cleveland, Ohio, USA; 4 Department of Pharmacology, Case Western Reserve University School of Medicine 12304, Cleveland, Ohio, USA; 5 Department of Biochemistry, Case Western Reserve University School of Medicine 12304, Cleveland, Ohio, USA; 6 Department of Proteomics and Bioinformatics, Case Western Reserve University School of Medicine, Cleveland, Ohio, USA; 7 CWRU-Cleveland VAMC Center for Antimicrobial Resistance and Epidemiology (Case VA CARES) 2546 , Cleveland, Ohio, USA; Instituto de Biologia Molecular y Celular de Rosario, Rosario, Santa Fe, Argentina

**Keywords:** artificial intelligence, antibiotic resistance, clinical microbiology

## LETTER

In the article “Confidence-based prediction of antibiotic resistance at the patient level” by Inda-Diaz et al., the authors present an extremely interesting, ambitious, and technically advanced study combining a transformer-based deep learning model with conditional inductive conformal prediction to estimate antimicrobial susceptibility from incomplete antimicrobial susceptibilty testing (AST) results and patient metadata (1). The scale of the data set—more than 12 million AST results from 1.6 million isolates across 30 European countries—is commendable and reflects substantial advancement in the field. The model’s performance across *Escherichia coli*, *Klebsiella pneumoniae*, and *Pseudomonas aeruginosa* for 16 antimicrobial agents, with an average F1 score of nearly 93%, underscores the significant progress being made in applying AI methods to clinical microbiology. In addition, the integration of an uncertainty estimation is a meaningful component, as it offers clinicians explicit guidance regarding prediction confidence. This is a necessary step forward. However, is it sufficient on its own to ensure clinical application?

Several points in the current study warrant more discussion. The pronounced variability in performance across antimicrobial classes—particularly the reduced accuracy for penicillins and aminoglycosides—raises some concerns about the consistency and clinical reliability of the model. Although the authors attribute these discrepancies to data imbalance and complex resistance mechanisms, these explanations require deeper examination. In these cases, perhaps clinical judgment must remain central, especially in scenarios where critical decisions must be made.

The exclusion of key organisms such as *Enterococcus* spp. and *Staphylococcus aureus* restricts the platform’s potential impact. Equally important is the omission of genomic data, which represents a missed opportunity to enhance predictive performance. As molecular diagnostics and sequencing become increasingly integrated into clinical workflows, models that disregard genotypic determinants risk falling behind current diagnostic paradigms.

Moreover, the paper raises the challenge associated with clinical deployment. Issues of clinician interaction with models such as this are not minor. They are central to whether such tools can be safely and effectively used. Without detailed “real-world validation” and implementation, even highly accurate systems remain concerning. Lastly, the performance of any AI/machine-learning model depends on the quality of the data used for training. In this study, the AST data of the large number of isolates were generated using multiple methods, such as Kirby-Bauer, Vitek, and Etest, which vary in consistency and interpretive reliability. Regional variation in microbiology, resistance prevalence, and the varying approaches of clinical microbiologists and infectious disease practitioners differ from location to location and from situation to situation.

In summary, the authors provide a promising and technically strong contribution to AI-enabled clinical microbiology. However, for this approach to achieve meaningful clinical impact, significant work remains. In the clinical arena, AI, intuition, and experience can go a long way to achieve successful therapy.
